# Correction: Relationship between the Relative Limitation and Resorption Efficiency of Nitrogen *vs* Phosphorus in Woody Plants

**DOI:** 10.1371/journal.pone.0094515

**Published:** 2014-04-16

**Authors:** The PLOS ONE Staff

## Notice of Republication

This article was republished on March 12, 2014, due to the omission of Equation 1. Please download this article again to view the correct version. The originally published, uncorrected article and the republished, corrected article are provided here for reference.

## Supporting Information

File S1 Originally published, uncorrected article.
(PDF)

File S2 Republished, corrected article.
(PDF)
