# Supplementary material for: Relationship between the Relative Limitation and Resorption Efficiency of Nitrogen vs Phosphorus in Woody Plants
Source: PLoS One. 2013 Dec 23;8(12):e83366. doi: 10.1371/journal.pone.0083366 (PMC3871644; doi:10.1371/journal.pone.0083366)
Supplement: Appendix S1 — Literature based on which the dataset were compiled for leaf nitrogen and phosphorus resorption efficiency in global woody plants. (PDF) [file pone.0083366.s001.pdf]

Appendix S1. Literature based on which the dataset were compiled for leaf nitrogen and phosphorus resorption efficiency in global woody plants.

1 Anderson, C.J. and Lockaby, B.G. 2011. Foliar nutrient dynamics in tidal and non-tidal freshwater forested wetlands. Aquatic Botany 95:153-160.

2 Berg, B., et al. 1996. Maximum decomposition limits of forest litter types: A synthesis. Canadian Journal of Botany-Revue Canadienne De Botanique 74:659-672.

3 Cai, Z.Q. and Bongers, F. 2007. Contrasting nitrogen and phosphorus resorption efficiencies in trees and lianas from a tropical montane rain forest in Xishuangbanna, south-west China. Journal of Tropical Ecology 23:115-118.

4 Chen, R.Y. 2011. Leaf N and P concentrations, and nutrient resorption efficiencies for five *Phyllostachys* bamboo species. Journal of Fujian College of Forestry 31:44-47.

5 Chen, X., et al. 1995. Comparative study on internal and external nutrient cyclings or poplar tree under different fertilizations □. Effect of fertilization on concentration and storage of major nutrients in poplar leaves before and after leaf fallen. Chinese Journal of applied ecology 6:346-348.

6 Covelo, F., et al. 2008. Spatial pattern and scale of leaf N and P resorption efficiency and proficiency in a *Quercus robur* population. Plant & Soil 311:109-119.

7 Delucia, E.H. and Schlesinger, W.H. 1995. Photosynthetic rates and nutrient-use efficiency among evergreen and deciduous shrubs in Okefenokee Swamp. International Journal of Plant Sciences 156:19-28.

8 Diehl, P., et al. 2003. Nutrient conservation strategies in native Andean-Patagonian forests. Journal of Vegetation Science 14:63-70.

9 Finzi, A.C., et al. 2001. Forest litter production, chemistry, and decomposition following two years of free-air CO_2 enrichment. Ecology 82:470-484.

10 Fioretto, A., et al. 2003. Litter-fall and litter decomposition in a low

Mediterranean shrubland. Biology and Fertility of Soils 39:37-44.

11 Freschet, G.T., et al. 2010. Substantial nutrient resorption from leaves, stems and roots in a subarctic flora: what is the link with other resource economics traits? New Phytologist 186:879-889.

12 Gallardo, J.F., et al. 1999. Nutrient efficiency and resorption in *Quercus pyrenaica* oak coppices under different rainfall regimes of the Sierra de Gata mountains (central western Spain). Annals of Forest Science 56:321-331.

13 Gerdol, R., et al. 2000. Water- and nutrient-use efficiency of a deciduous species, *Vaccinium myrtillus*, and an evergreen species, *V. vitis-idaea*, in a subalpine dwarf shrub heath in the southern Alps, Italy. Oikos 88:19-32.

14 Gillon, D., et al. 1999. Nitrogen and phosphorus cycling following prescribed burning in natural and managed Aleppo pine forests. Canadian Journal of Forest Research-Revue Canadienne De Recherche Forestiere 29:1237-1247.

15 Guo, F. and Zhou, Y.C. 2010. Foliar nutrient contents and translocation features in different density of *Pinus massoniana* plantations. Journal of Nanjing Forestry University 34:93-96.

16 He, H., et al. 2011. Dinitrogen-fixing *Acacia* species from phosphorus-impoverished soils resorb leaf phosphorus efficiently. Plant, Cell & Environment 34:2060-2070.

17 Hevia, F., et al. 1999. Foliar nitrogen and phosphorus dynamics of three *Chilean nothofagus* (Fagaceae) species in relation to leaf lifespan. American Journal of Botany 86:447-455.

18 Hobbie, S.E. 2005. Contrasting effects of substrate and fertilizer nitrogen on the early stages of litter decomposition. Ecosystems 8:644-656.

19 Inagaki, M., et al. 2011. Nitrogen and phosphorus retranslocation and N:P ratios of litterfall in three tropical plantations: luxurious N and efficient P use by *Acacia mangium*. Plant & Soil 341:295-307.

20 Killingbeck, K. 1993. Inefficient nitrogen resorption in genets of the actinorhizal nitrogen fixing shrub *Comptonia eregrina*: physiological ineptitude

or evolutionary tradeoff? Oecologia 94:542-549.

21 Killingbeck, K.T. 1984. Nitrogen and phosphorus resorption dynamics of five tree species in a Kansas gallery forest . American Midland Naturalist 111:155-164.

22 Killingbeck, K.T. and Whitford, W.G. 2001. Nutrient resorption in shrubs growing by design, and by default in Chihuahuan Desert arroyos. Oecologia 128:351-359.

23 Lal, C.B., et al. 2001. Foliar demand and resource economy of nutrients in dry tropical forest species. Journal of Vegetation Science 12:5-14.

24 Lee, D.W., et al. 2003. Pigment dynamics and autumn leaf senescence in a New England deciduous forest, eastern USA. Ecological Research 18:677-694.

25 Lindsay, E.A. and French, K. 2005. Litterfall and nitrogen cycling following invasion by *Chrysanthemoides monilifera* ssp *rotundata* in coastal Australia. Journal of Applied Ecology 42:556-566.

26 Liu, B., et al. 2010. Seasonal variation and resorption characteristics of leaf N,P,and K in two aged *Xanthoceras sorbifolia* plantations. Chinese Journal of Ecology 29:1270- 1276.

27 Medina, E., et al. 2010. Nutrient relations of dwarf *Rhizophora mangle* L. mangroves on peat in eastern Puerto Rico. Plant Ecology 207:13-24.

28 Polglase, P.J. and Attiwill, P.M. 1992. Nitrogen and phosphorus cycling in relation to stand age of *eucalyptus regnans* F. Muell. I. Return from plant to soil in litterfall. Plant & Soil 142:157-166.

29 Reich, P.B., et al. 1995. Leaf carbon and nutrient assimilation and conservation in species of differing successional status in an oligotrophic Amazonian forest. Functional Ecology 9:65-76.

30 Scott, D.A., et al. 1992. Ecological-studies on a lowland evergreen rain-forest on Maraca Island, Roraima, Brazil. II. Litter and nutrient cycling. Journal of Ecology 80:705-717.

31 Sundqvist, M.K., et al. 2011. Within- and across-species responses of plant

traits and litter decomposition to elevation across contrasting vegetation types in subarctic tundra. PLoS ONE 6:e27056.

32 Vitousek, P.M. 1998. Foliar and litter nutrients, nutrient resorption, and decomposition in Hawaiian *Metrosideros polymorpha*. Ecosystems 1:401-407.

33 Wang, W., et al. 2011. Influence of frost on nutrient resorption during leaf senescence in a mangrove at its latitudinal limit of distribution. Plant & Soil 342:105-115.

34 Wang, X.H., et al. 2004. A study on leaf nutrient resorption of some trees in Tiantong national forest park. Guihaia 24:81 - 85.

35 Wardle, D.A., et al. 2009. Indirect effects of invasive predators on litter decomposition and nutrient resorption on seabird-dominated islands. Ecology 90:452-464.

36 Xu, F.Y., et al. 1997. Internal and external nutrient transfers in foliage of some north deciduous trees. I. Changes of nutrient concentrations and contents. Chinese Journal of applied ecology 8:1-6.

37 Xue, L. and Luo, S. 2003. Changes in nitrogen and phosphorus and their retranslocation in leaves of evergreen and deciduous broadleaved trees. Forest Research 16:166-170.

38 Xue, L., et al. 2005. Seasonal patterns in nitrogen and phosphorus and resorption in leaves of four tree species. Acta Ecologica Sinica 25:520-526.

39 Yin, Y. and Lin, P. 1993. The accumulation and biological cycle of nitrogen and phosphrus elements in *Rhizophors stylosa* community, Guangxi. Acta Ecologica Sinica 13:221-227.

40 Zhuang, Y.Z. 2010. Content and retranslocation of nutrient in needlelike leaves of *Pinus massoniana* at different elevations. Modern Agricultural Sciences & Technology 20:219-220.
