# Supplementary material for: Relationship between the Relative Limitation and Resorption Efficiency of Nitrogen vs Phosphorus in Woody Plants
Source: PLoS One. 2013 Dec 23;8(12):e83366. doi: 10.1371/journal.pone.0083366 (PMC3871644; doi:10.1371/journal.pone.0083366)
Supplement: Figure S1 — Global distribution of green-leaf N:P ratio and the relative resorption efficiency. (PDF) [file pone.0083366.s002.pdf]

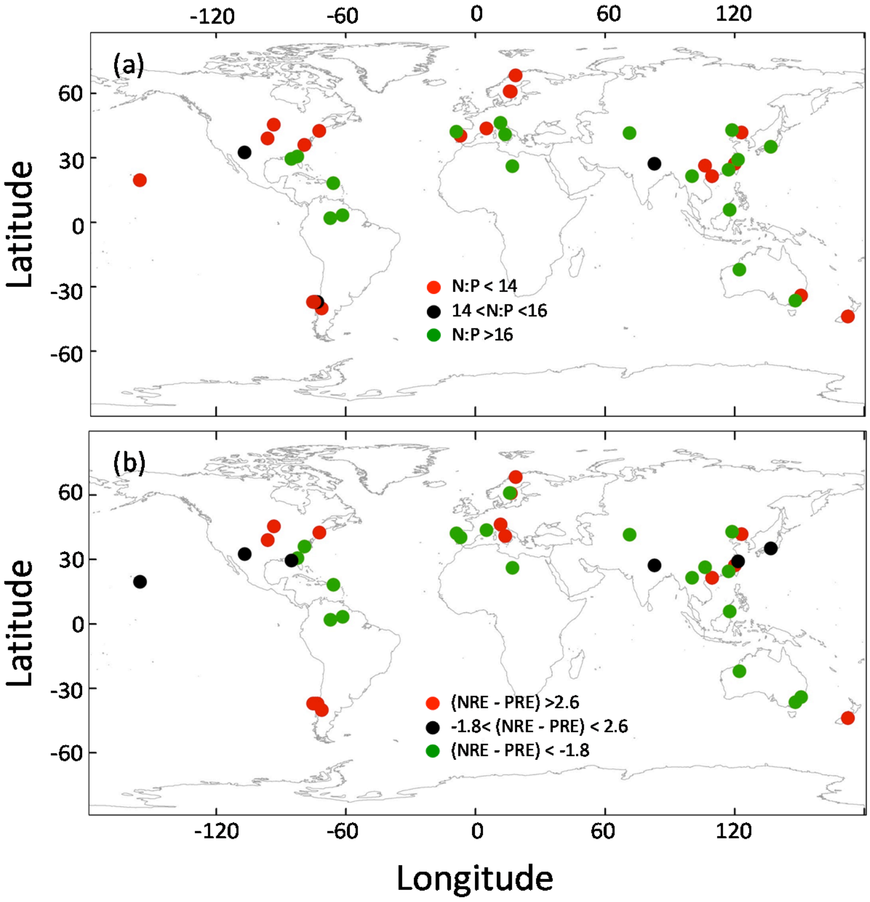

Figure S1. Global distribution of (a) green-leaf N:P ratio and (b) the relative resorption efficiency (NRE - PRE) in this study. The thresholds (2.6 and -1.8) of (NRE - PRE) were calculated according to the regression model in Figure 1a, corresponding to the N:P thresholds 14 and 16, respectively. Each data point represents a site-averaged value.
