# Supplementary material for: Relationship between the Relative Limitation and Resorption Efficiency of Nitrogen vs Phosphorus in Woody Plants
Source: PLoS One. 2013 Dec 23;8(12):e83366. doi: 10.1371/journal.pone.0083366 (PMC3871644; doi:10.1371/journal.pone.0083366)
Supplement: Figure S2 — Relationships between relative resorption efficiency and the latitude/climates. (PDF) [file pone.0083366.s003.pdf]

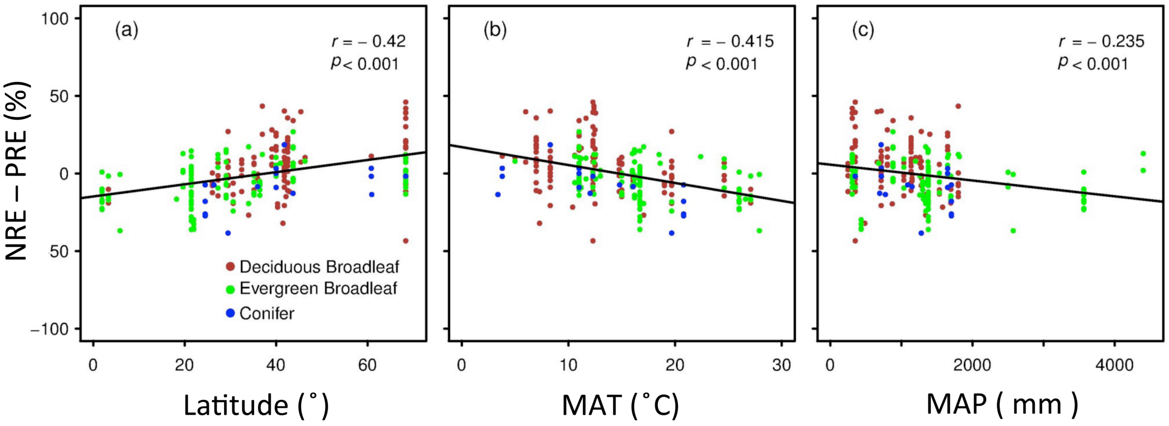

Figure S2. The relationship between relative resorption efficiency (NRE - PRE) and (a) the absolute latitude (°), (b) mean annual temperature (MAT, °C) and (c) mean annual precipitation (MAP, mm). All the lines were fitted with ordinary least square linear regression.
