# Supplementary material for: Relationship between the Relative Limitation and Resorption Efficiency of Nitrogen vs Phosphorus in Woody Plants
Source: PLoS One. 2013 Dec 23;8(12):e83366. doi: 10.1371/journal.pone.0083366 (PMC3871644; doi:10.1371/journal.pone.0083366)
Supplement: Table S1 — Uncorrected nutrient resorption efficiency and senesced-leaf N and P concentration for different plant types. (PDF) [file pone.0083366.s004.pdf]

Table S1. Uncorrected NRE/PRE and N_{sen}/P_{sen} for different plant types.

		**NRE (%)**		**PRE (%)**		**N:P ratio**		**N_{sen} (mg g^{-1})**		**P_{sen} (mg g^{-1})**	
	n	Mean	SE	Mean	SE	Mean	SE	Mean	SE	Mean	SE
**Leaf habit**											
DB	110	50.0^{aA}	1.8	42.5^{aB}	2.2	13.5^{a}	0.5	9.8^{a}	0.4	1.04^{a}	0.07
EB	113	37.0^{bA}	1.9	44.2^{aB}	2.3	18.0^{b}	0.8	10.7^{a}	0.5	0.66^{b}	0.05
Conifer	16	48.1^{aA}	3.1	60.3^{bB}	2.9	12.0^{a}	1.1	5.2^{b}	0.5	0.40^{b}	0.07
**N-fixer**											
Yes	19	31.3^{aA}	3.9	53.7^{aB}	5.0	22.9^{a}	3.2	15.4^{a}	0.9	0.68^{a}	0.14
No	220	44.8^{bA}	1.4	43.7^{bA}	1.6	14.8^{b}	0.4	9.4^{b}	0.3	0.82^{a}	0.05
**Overall**	239	43.7^{A}	1.3	44.5^{A}	1.5	15.4	0.5	9.9	0.3	0.81	0.04

Number of replicates (site×species, *n*), mean value and its standard error (SE) are reported. Differences in the variables between each type are tested using ANOVA and *t*-test with Bonferroni corrections. Different letters indicate significant differences in variables ($p < 0.05$) between comparisons: small letters (a/b/c) for DB *vs* EB *vs* conifers, or N-fixers *vs* non-N-fixers; and captical letters (A/B) for NRE *vs* PRE. Nitrogen and phosphorus nutrient resorption efficiency (NRE/PRE), and senesced-leaf nitrogen (N_{sen}) and phosphorus (P_{sen}) are not corrected with leaf mass loss (see the Methods for details). DB, deciduous broadleaf; EB, evergreen broadleaf. Green-leaf N:P ratios (N:Pgr) were also shown.
