# Supplementary material for: Relationship between the Relative Limitation and Resorption Efficiency of Nitrogen vs Phosphorus in Woody Plants
Source: PLoS One. 2013 Dec 23;8(12):e83366. doi: 10.1371/journal.pone.0083366 (PMC3871644; doi:10.1371/journal.pone.0083366)
Supplement: Table S2 — Results of fertilization experiments with conifer vegetation, showing green-leaf N:P ratios and nutrient status of the vegetation. (PDF) [file pone.0083366.s005.pdf]

Table S2. Results of fertilization experiments with conifer vegetation, showing green-leaf N:P ratios and nutrient status of the vegetation.

N:P	Limitation	Vegetation	Fertilization	Location
7.54	N	*Picea abies* stand	N & P	Sweden
8	N	Douglas fir forest	N & P	The Netherlands
8.96	N	*Pinus sylvestris* stand	N & P	Sweden
7.0-14.5	Not N	Coniferous forest	N	Europe
9.8	N & P	*Picea abies* plantation	N & P	Sweden
10.42	N & P	Loblolly pine plantation	N & P	North Carolina

Re-organized according to Tessier & Raynal (2003) [22].
