# Supplementary material for: Correction: Relationship between the Relative Limitation and Resorption Efficiency of Nitrogen vs Phosphorus in Woody Plants
Source: PLoS One. 2014 Apr 16;9(4):e94515. doi: 10.1371/journal.pone.0094515 (PMC3989191; doi:10.1371/journal.pone.0094515)
Supplement: File S2 — Republished, corrected article. (PDF) [file pone.0094515.s002.pdf]

# Relationship between the Relative Limitation and Resorption Efficiency of Nitrogen *vs* Phosphorus in Woody Plants

**Wenxuan Han[1*], Luying Tang[2], Yahan Chen[3], Jingyun Fang[2*]**

**1** Key Laboratory of Plant-Soil Interactions, Ministry of Education, College of Resources and Environmental Sciences, China Agricultural University, Beijing, China, **2** Key Laboratory for Earth Surface Processes, Ministry of Education, Department of Ecology, Peking University, Beijing, China, **3** Institute of Botany, Chinese Academy of Sciences, Beijing, China

## Abstract

Most previous studies have ascribed variations in the resorption of a certain plant nutrient to its corresponding environmental availability or level in tissues, regardless of the other nutrients' status. However, given that plant growth relies on both sufficient and balanced nutrient supply, the nutrient resorption process should not only be related to the absolute nutrient status, but also be regulated by the relative limitation of the nutrient. Here, based on a global woody-plants dataset from literature, we test the hypothesis that plants resorb proportionately more nitrogen (or phosphorus) when they are nitrogen (or phosphorus) limited, or similar proportions of nitrogen (N) and phosphorus (P) when co-limited by both nutrients (the relative resorption hypothesis). Using the N:P ratio in green foliage as an indicator of nutrient limitation, we found an inverse relationship between the difference in the proportionate resorption of N *vs* P and this foliar N:P ratio, consistent across species, growth-forms, and vegetation-types globally. Moreover, according to the relative resorption hypothesis, communities with higher/lower foliar N:P (more likely P/N limited) tend to produce litter with disproportionately higher/lower N:P, causing a worsening status of P/N availability; this positive feedback may somehow be counteracted by several negative-feedback mechanisms. Compared to N, P generally shows higher variability in resorption efficiency (proportion resorbed), and higher resorption sensitivity to nutrient availability, implying that the resorption of P seems more important for plant nutrient conservation and N:P stoichiometry. Our findings elucidate the nutrient limitation effects on resorption efficiency in woody plants at the global scale, and thus can improve the understanding of nutrient resorption process in plants. This study also suggests the importance of the foliar N:P ratio as a key parameter for biogeochemical modeling, and the relative resorption hypothesis used to deduce the critical (optimal) N:P ratio for a specific plant community.

**Citation:** Han W, Tang L, Chen Y, Fang J (2013) Relationship between the Relative Limitation and Resorption Efficiency of Nitrogen *vs* Phosphorus in Woody Plants. PLoS ONE 8(12): e83366. doi:10.1371/journal.pone.0083366

**Editor:** Randall P. Niedz, United States Department of Agriculture, United States of America

**Received** July 23, 2013; **Accepted** November 1, 2013; **Published** December 23, 2013

**Copyright:** © 2013 Han et al. This is an open-access article distributed under the terms of the Creative Commons Attribution License, which permits unrestricted use, distribution, and reproduction in any medium, provided the original author and source are credited.

**Funding:** The research was funded by National Natural Science Foundation of China (41173083, 31021001), and National Basic Research Program of China on Global Change (2010CB950600).The funders had no role in study design, data collection and analysis, decision to publish, or preparation of the manuscript.

**Competing interests:** The authors have declared that no competing interests exist.

* E-mail: hanwenxuan@cau.edu.cn (WH); jyfang@urban.pku.edu.cn (JF)

## Introduction

Plants have evolved diverse strategies to overcome nutrient shortages, or the relative limitations caused by an unbalanced environmental nutrient supply [1]. Nutrient resorption is one key strategy [2-4]: perennial plants withdraw nutrients from senescing leaves and re-translocate and store them in the stem- and root-pools. The resorbed nutrients then later can be reused to build new tissues (such as leaves or seeds) with relatively less cost (energy and time) than absorbing nutrients from the soil [5].

Because of its importance in nutrient conservation and in biogeochemical cycling at the ecosystem level, nutrient resorption processes have been intensely studied from leaf to global scales [4-11], General patterns and the underlying mechanisms governing resorption strategies, however, have been elusive. There is considerable variation in nutrient resorption efficiency (NuRE: the proportion of nutrient withdrawn before leaf abscission) for foliar nitrogen (N) *vs* phosphorus (P) as well as between plant groups (e.g., evergreen *vs* deciduous woody plants and angiosperms *vs* conifers) [5,6,12,13]. There traditionally has been a plausible idea that environmental nutrient availability will influence NuRE [14]; this nutritional control viewpoint supposes that plants growing in low fertility environments (e.g., conifers, and tropical evergreens) should have higher NuRE than those with high

nutrient supply rates. Although plants in infertile environments usually do have longer leaf lifespan and lower leaf nutrient concentration, the negative relationship between nutrient availability and NuRE has not proved ubiquitous [8,15]. For example, evergreens, which are often dominant in infertile environments, show similar or even lower nitrogen or phosphorus resorption efficiency (NRE or PRE) than deciduous plants on nutrient-rich soils [14]. Moreover, fertilization (N/P addition) experiments seemed to suggest that nutrient supply had inconsistent, if any, impact on NuRE [6,14,16].

Nevertheless, several recently observed large-scale patterns of NuRE based on literature data or fertilization experiments [9-11,17-19] do suggest the existence of nutritional control on NuRE at least on a biogeographical scale. Significant trends in NuRE along latitudinal, climatic and edaphic gradients have been observed at regional [18,19] and global scales [9-11]. Since plant growth relies on a sufficient as well as balanced nutrient supply (Liebig's law of the minimum [1]), the nutrient resorption process should not only be related to the nutrient status (e.g., green-leaf N/P) [8,10], but also be regulated by the nutrient stoichiometry (N:P ratio of nutrient availability) [7,20]. Using foliar N:P as the indicator to identify the limiting nutrient (N *vs* P) [6,7,21-23], we hypothesize that if a plant is under a balanced growth, it should have a roughly balanced nitrogen versus phosphorus resorption efficiency (NRE ≈ PRE), while if there is N (or P) limitation, the plant will resorb a greater (or smaller) proportion of N compared to P (NRE > PRE, or NRE < PRE, respectively). Briefly, the hypothesis supposes that plants tend to resorb a greater proportion of nutrients limiting their growth, termed here the "relative resorption hypothesis". In this study, we will test this hypothesis using a global dataset of leaf nutrient resorption in woody plants.

## Materials and Methods

We compiled data from literature reporting pair-wised concentration of N and P in both fully expanded green and senesced leaves lumped by species within a site. For studies reporting N and P concentrations as a time series over the growing season or longer, we used the maximum value of mature leaves for green-leaf nutrient concentration. For data from fertilization studies, we used only data from unfertilized controls. All these published papers were gathered using Web of Science, Google Scholar and Chinese Journal Net search engines and the following key words: resorption, reabsorption, retranslocation, nutrient use efficiency, and their Chinese counterparts. The references in the studies we retrieved were also examined. Overall, our database was drawn from 40 studies (Appendix S1) encompassing 199 woody species, grouped into three growth forms: deciduous broadleaf (DB) species, evergreen broadleaf (EB) species, and conifers. Note that in our dataset all the conifers are evergreen except *Larix kaempferi*. We also noted and compared nutrient concentration and resorption efficiency between species that fix or do not fix nitrogen. Data originate from sites on every continent except Antarctica, from 43°42′ S to 68°21′N (Figure S1), from 3.4 °C to 27.9 °C mean annual temperature (MAT) regimes, and from 250 mm to 4400 mm mean annual precipitation (MAP) regimes (Figure S2). Types of the vegetation from which leaves were sampled were also recorded according to the literature.

Nutrient resorption efficiency (NuRE) , the proportional withdrawal of nutrients during leaf senescence, was expressed as [8,14]:

$$\mathrm{NuRE} = \frac{\mathrm{Nu_{green}\text{-}Nu_{senesced}}}{\mathrm{Nu_{green}}} \times 100\% \quad \textbf{(1)}$$

where $Nu_{green}$ and $Nu_{senesced}$ are nutrient concentration (N and P) in green and senesced leaves, respectively.

Considering the leaf mass loss when a leaf senesces [24], we used a mass loss correction factor (MLCF) to compensate the underestimation of NuRE. In this study, senesced-leaf nutrient concentration and NuRE for analysis were corrected, using Eq. (2) and Eq. (3):

$$\mathrm{Nu'_{senesced}} = Nu_{senesced} \times MLCF \quad \textbf{(2)}$$

$$\mathrm{NuRE} = \frac{(\mathrm{Nu_{green}\text{-}Nu'_{senesced}})}{\mathrm{Nu_{green}}} \times 100\% \quad \textbf{(3)}$$

where $Nu'_{senesced}$ is the $Nu_{senesced}$ corrected with MLCF. The MLCFs were different across growth forms: 0.784 for deciduous broadleaves, 0.780 for evergreen broadleaves, and 0.745 for conifers [10]. However, to facilitate comparison with previous studies, the uncorrected $Nu_{senesced}$ and NuRE were also reported (Table S1).

We used standardized major axis (SMA) regression analysis (type II regression) to characterize the scaling relationship between variables, given that both variables were measured with error [25]. We used the difference between NRE and PRE (NRE - PRE) to indicate the relative resorption proportion of N *vs* P, and the green-leaf N:P mass ratio (N:Pgr, or foliar N:P) to indicate the relative limitation of nutrient (N *vs* P) to plants. Statistical analyses were performed using R 2.14.2 [26].

## Results

### Variation in leaf N/P and NRE/PRE

The overall means of NRE and PRE were 56.3% and 56.9% (mass-loss corrected), or 43.7% and 44.5% (uncorrected), respectively (Table 1; Table S1). The average green-leaf N:P mass ratio was 15.4. There was no significant difference between the overall average NRE and PRE ($p$ = 0.36), but for different plant types (DB, EB, conifer, and N-fixing species), NRE was significantly different from PRE ($p < 0.05$). The CV of PRE was larger than that of NRE for overall plants (33% *vs* 28%), and for different plant types except for conifer.

Evergreen broadleaves showed significantly lower NRE than conifers (50.1% *vs* 61.0%) and deciduous species (61.2%), while conifers had higher PRE than angiosperms (70% *vs* 56%) (all $p < 0.05$). Nitrogen fixing plants displayed significantly lower NRE (46.3% *vs* 57.2%), but higher PRE than non-N-fixers (63.8% *vs* 56.3%) ($p < 0.05$).

**Table 1.** Nitrogen and phosphorus resorption efficiency (NRE/PRE), green-leaf N:P, senesced-leaf nitrogen ($N_{sen}$) and phosphorus ($P_{sen}$) for different plant types.

| | | NRE (%) | | PRE (%) | | N:P ratio | | $N_{sen}$ (mg $g^{-1}$) | | $P_{sen}$ (mg $g^{-1}$) | |
|---|---|---|---|---|---|---|---|---|---|---|---|
| | *n* | **Mean** | **SE** | **Mean** | **SE** | **Mean** | **SE** | **Mean** | **SE** | **Mean** | **SE** |
| **Leaf habit** | | | | | | | | | | | |
| DB | 110 | $61.2^{aA}$ | 1.3 | $55.3^{aB}$ | 1.7 | $13.5^{a}$ | 0.5 | $7.7^{a}$ | 0.3 | $0.81^{a}$ | 0.05 |
| EB | 113 | $50.1^{bA}$ | 1.5 | $56.5^{aB}$ | 1.8 | $18.0^{b}$ | 0.8 | $8.3^{a}$ | 0.4 | $0.51^{b}$ | 0.04 |
| Conifer | 16 | $61.0^{aA}$ | 2.5 | $70.3^{bB}$ | 2.2 | $12.0^{a}$ | 1.1 | $3.9^{b}$ | 0.3 | $0.30^{b}$ | 0.05 |
| **N-fixer** | | | | | | | | | | | |
| Yes | 19 | $46.3^{aA}$ | 3.0 | $63.8^{aB}$ | 3.9 | $22.9^{a}$ | 3.2 | $12.0^{a}$ | 0.7 | $0.68^{a}$ | 0.14 |
| No | 220 | $57.2^{bA}$ | 1.0 | $56.3^{bA}$ | 1.2 | $14.8^{b}$ | 0.4 | $7.4^{b}$ | 0.2 | $0.82^{a}$ | 0.05 |
| **Overall** | 239 | $56.3^{A}$ | 1.0 | $56.9^{A}$ | 1.2 | 15.4 | 0.5 | 7.8 | 0.2 | 0.81 | 0.04 |

Number of replicates (site×species, *n*), mean value and its standard error (SE) are reported. Differences in the variables are tested using ANOVA and *t*-test with Bonferroni corrections. Different letters indicate significant differences in variables ($p < 0.05$) between comparisons: small letters (a/b/c) for DB *vs* EB *vs* conifers, or N-fixers *vs* non-N-fixers; and capital letters (A/B) for NRE *vs* PRE. NRE/PRE and $N_{sen}/P_{sen}$ have been corrected with leaf mass loss (see the Methods for details). DB, deciduous broadleaf; EB, evergreen broadleaf.

doi: 10.1371/journal.pone.0083366.t001

### Relationship between Green-Leaf N:P Ratio and NRE/PRE

The relative resorption efficiency (NRE - PRE) was significantly negatively correlated to green-leaf N:P ratio ( $r^2$ = 0.20, $p$ < 0.0001) across all plants (Figure 1a). The inverse relationship also existed for plants with different leaf habits ($r^2$ = 0.14, 0.25, and 0.30, and $p$ < 0.0001, 0.0001, and 0.05 for DB, EB and conifer, respectively; Figure 1b-d), although it was only marginally significant for N-fixers ($r^2$ = 0.20; $p$ = 0.053; Figure 1d).

The horizontal dash lines indicate where NRE equals PRE, and the vertical dash lines display the corresponding N:P ratios.

The N:Pgr ratio was 15.2 corresponding to NRE equal to PRE for all plants, and 15.4, 14.3, 9.0, and 8.0 for DB, EB, conifers and N-fixers, respectively, according to the type II regression models (Figure 1).

NRE showed significantly negative correlation with green-leaf N:P ratio for overall species ($r^2$ = 0.02, $p$ = 0.016) and for BD ($r^2$ = 0.04, $p$ = 0.038) (Figure 2a-b). While PRE was significantly positively correlated to green-leaf N:P ratio for overall species and for each plant type ($r^2$ = 0.07, 0.04, 0.13 and 0.29 for all species, DB, EB and conifers, respectively; all $p$ < 0.05; Figure 2). Moreover, the NRE slopes of SMA regression are generally shallower than PRE slopes: 2.1 *vs* 2.5 for all species, 2.5 *vs* 3.1 for DB, and non-significantly different from zero ($p$ = 0.76 and 0.27) *vs* 2.3 and 1.9 for EB and conifers, respectively.

The blue symbols and lines denote NRE, and the red ones represent PRE. Regression lines of NRE *vs* N:P are not shown in panel (c) and (d) for lack of significant correlations between the two variables in EB and conifers ($p$ = 0.76 and 0.27, respectively).

### Relationship between green-leaf N:P and senesced-leaf N and P

There were significant positive correlations between green- and senesced-leaf N:P in each plant type and in all species ($r^2$ = 0.65, 0.80, 0.77, and 0.73 for DB, EB, conifers, and overall plants, respectively; all $p$ < 0.0001). All the type II regression slope estimates were significantly greater than 1.0 (1.47~1.73) (Figure 3). There were significant correlations between green-leaf N:P ratio and senesced-leaf nutrients (positive for N and negative for P) for all species ($r^2$ = 0.06 and 0.44 for N and P, respectively; both $p$ < 0.0001; Figure 4).

## Discussion

### Patterns of NRE and PRE and NRE *vs* PRE

Our results showed that woody plants resorbed similar proportions of N and P before leaf abscission (Table 1). The mass-loss corrected overall mean NRE and PRE (56% and 57%) are comparable to those reported in previous studies (e.g., 57% and 55% [10]), and the corresponding uncorrected ones (44% and 44.5%) (Table S1) are comparable to those (e.g., 50.5% and 51% [14], 47% and 54% [9]) reported for woody plants.

Among the three woody plant groups with contrasting leaf habits, the NRE are lowest in EB while the PRE are highest in conifers (Table 1). This variation pattern across growth-forms is different from that reported by some previous studies [9,10], but similar to that reported by others [14].

In our study, the NRE is lower in EB, conifers, and N-fixers, but higher in DB, compared to the corresponding PRE in the respective plant type (Table 1). The pattern of NRE *vs* PRE is consistent with that reported in reference [14], but different from that in reference [9]. These inconsistent observations on variations in NuRE will be further discussed afterwards using our model proposed in this study (the relative resorption hypothesis).

Interestingly, the variation in PRE is generally larger than that in NRE (1.2~3.9 *vs* 1.0~3.0 for SE of PRE and NRE, respectively; Table 1), consistent with that observed by Aerts & Chapin (2000) [6]. Given that part of the leaf N (e.g., N in cell walls) is immobile while most of the P is hydrolysable and can be resorbed [12,17], the larger variation in PRE may reflect more sensible response of plant P to the environments [19,27-30]) and larger variability of environmental (soil) P on the earth because of its rock-origin [17,31], compared to the biology-derived N.

### Senesced-Leaf N and P Concentration and Its Relationship with Green-Leaf N:P

Killingbeck (1996) [4] argued that the nutrient concentrations in senesced leaves (nutrient resportion proficiency, NuRP) is a result of natural selection acting to achieve an optimum resorption for plant nutrients. Thus, according to the NuRP thresholds proposed by Killingbeck (1996) [4] (see Figure 4 for

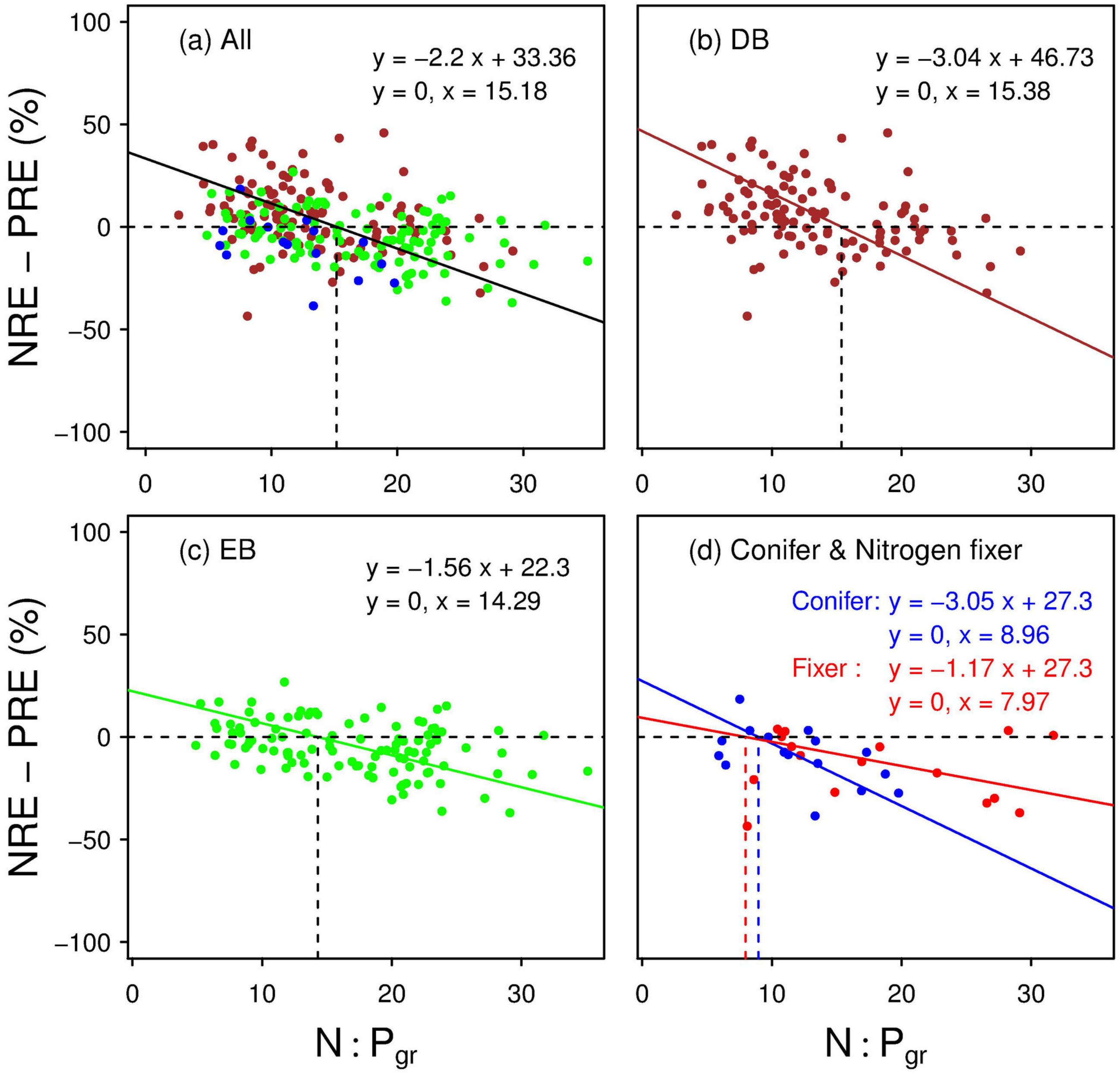


**Figure 1. Standardized major axis regression (solid lines) for relative resorption efficiency (NRE - PRE) vs green-leaf N:P ratio for (a) all species, black lines; (b) deciduous broadleaf species (DB), brown dots and lines; (c) evergreen broadleaf (EB), green dots and lines; (d) conifers and N-fixing species, respectively blue and red dots and lines.** The horizontal dash lines indicate where NRE equals PRE, and the vertical dash lines display the corresponding N:P ratios.

doi: 10.1371/journal.pone.0083366.g001

details), plants tend to reach a complete N resorption but incomplete P resorption when they suffer from N-limitation (e.g., N:P < 10), or reach a complete P resorption but incomplete N resorption when they are under P-limitation (e.g., N:P > 20). When plants are under N/P balanced growth (co-limited by N and P, e.g., N:P = 15 for angiosperms), they can only obtain an incomplete or intermediate resorption for both N and P (Figure 4).

### Linkage between the relative limitation and resorption of N *vs* P

Some recent studies showed evidence that nutrient availability would influence NRE/PRE at large scales [17-19]. For example, in experiments with phosphorus and nitrogen addition in mangrove forests over 30 degrees of latitude, Lovelock et al. (2007) [19] found that PRE decreased with the gradient of soil P availability, and NRE decreased with

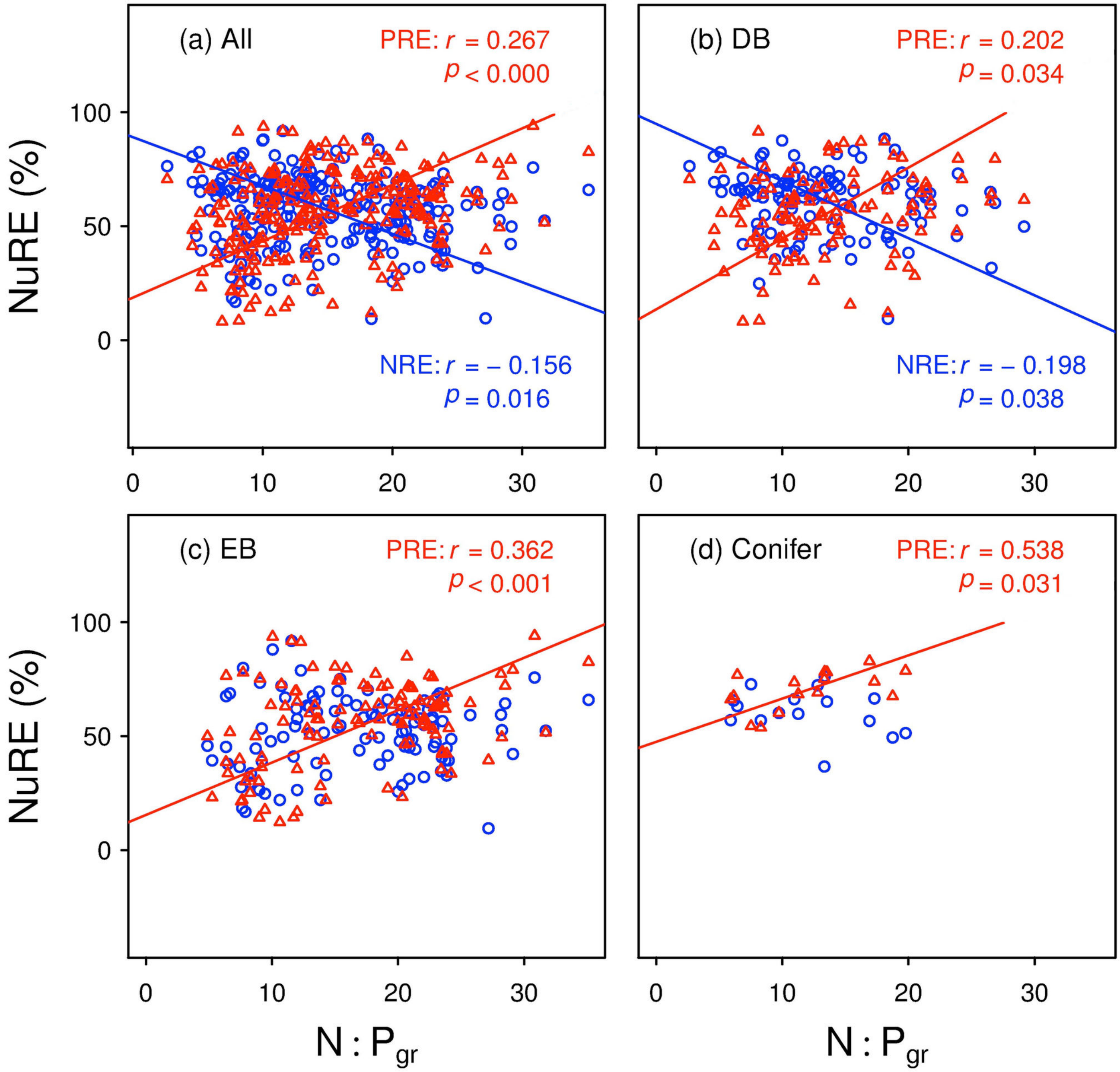


**Figure 2. Standardized major axis regression for N or P resorption efficiency (NRE or PRE, NuRE) *vs* green-leaf N:P ratio for (a) all species; (b) deciduous broadleaf species (DB); (c) evergreen broadleaf species (EB); and (d) conifers.** The blue symbols and lines denote NRE, and the red ones represent PRE. Regression lines of NRE vs N:P are not shown in panel (c) and (d) for lack of significant correlations between the two variables in EB and conifers ($p$ = 0.76 and 0.27, respectively).

doi: 10.1371/journal.pone.0083366.g002

increasing N availability. An experiment along a soil chronosequence also suggested a negative response of PRE to soil P gradient [18]. We therefore assumed that the relative resorption efficiency of N *vs* P (indicated by the difference in resorption efficiency between N and P, i.e., NRE minus PRE) could be controlled by the relative N/P limitation and reflected in the foliar N:P ratio.

We did find a negative relationship between (NRE-PRE) and green-leaf N:P (Figure 1). In contrast, correlations between NRE/PRE and N:Pgr are very weak or non-significant (Figure 2). This is consistent with the observation in two graminoid wetlands by Güsewell (2005) [7], who suggested that resorption efficiency should not be directly adjusted to the type of nutrient limitation. Güsewell (2005) [7] found that N:P ratios of senesced leaves were always higher than N:P ratios of

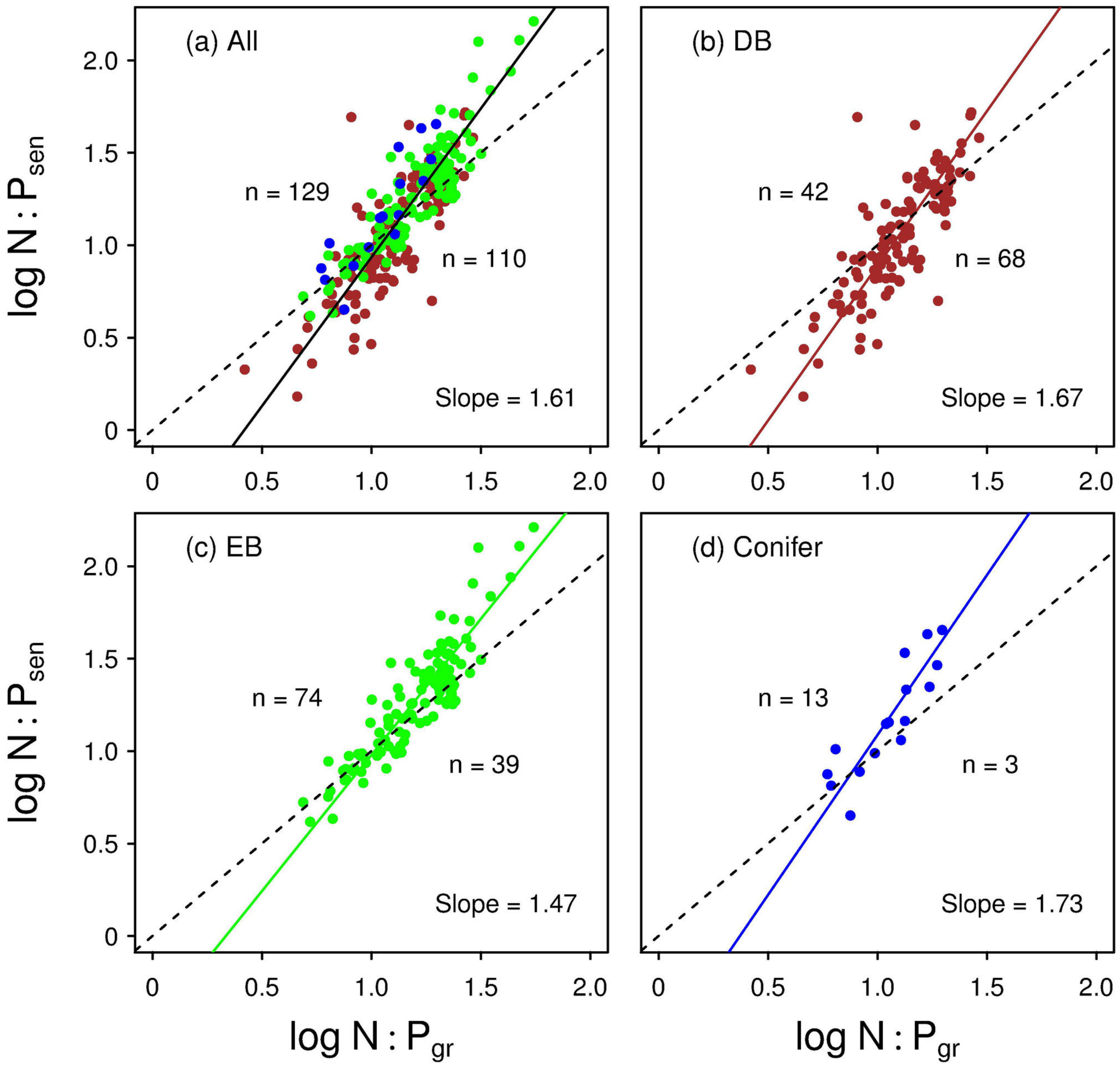


**Figure 3. Standardized major axis regression (solid lines) for $log_{10}$-transfromed N:P ratio in senesced against green leaves of (a) all species, (b) deciduous (DB) and (c) evergreen (EB) angiosperms, and (d) conifers.** The dash lines denote the 1:1 lines, above and below which the numbers of data points are shown.

doi: 10.1371/journal.pone.0083366.g003

green leaves, while N:P resorption ratios were always smaller than one, regardless of the N:P ratios of green leaves. The different conclusions between reference [7] and this study seem to suggest the difference in the regime of NuRE between wetland graminoids and woody plants. Thus, the relative resorption efficiency (NRE-PRE) might better mirror the evolutionary significance of the resorption process in plants, compared to NRE or PRE separately.

The difference in resorption efficiency between N and P (NRE-PRE) declined with increasing N:Pgr ratio, which is consistent with the prediction of our hypothesis. The woody plants as a whole reached a balanced resorption efficiency (NRE = PRE) around a ratio of N:Pgr = 15, which suggests an overall N/P balanced plant growth. This critical value of N:Pgr ratio is just the mid-point within the range of N and P co-limited growth (e.g., 10~20 for terrestrial plants [7]; or, 14~16 for wetland plants [21]). When green-leaf N:P decreased to less

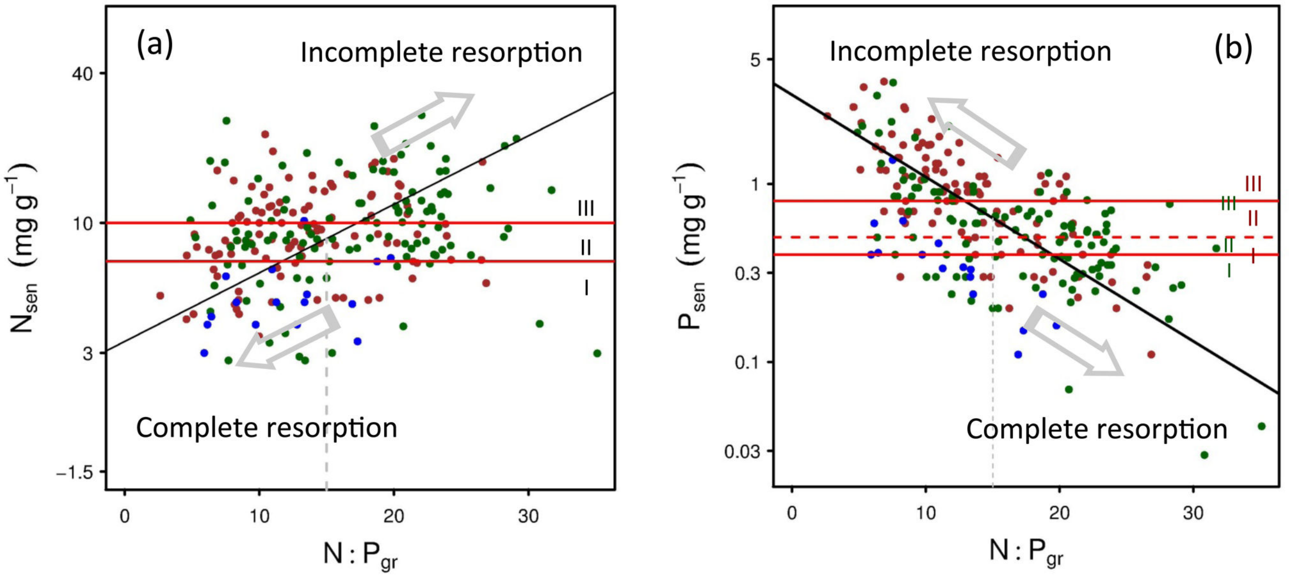


**Figure 4. Relationship between senesced-leaf N/P (uncorrected, in log-scale) and green-leaf N:P ratio.** The three zones (I/II/III, delimitated by horizontal red lines) of senesced-leaf N and P [4] are: < 7 mg $g^{-1}$ (zone I, complete resorption) and > 10 mg $g^{-1}$ (zone III, incomplete resorption) for N (a); < 0.5 mg $g^{-1}$ or < 0.4 mg $g^{-1}$ (zone I, complete resorption for deciduous and evergreen species, respectively) and > 0.8 mg $g^{-1}$ or > 0.5 mg $g^{-1}$ (zone III, incomplete resorption for deciduous and evergreen species, respectively) for P (b). Zones II are intermediate resorption ranges in both panels. The red and green points and Roman numbers indicate the data and zones for deciduous and evergreen species, respectively. The blue points denoting conifers were also shown.

doi: 10.1371/journal.pone.0083366.g004

than the critical ratio (plants tend to be N-limited), (NRE-PRE) increased above zero, which meant PRE decreases and/or NRE increases accordingly (Figure 2). On the contrary, when N:Pgr ratio increased to greater than the critical value (plants tend to be P-limited), then (NRE-PRE) took negative values.

Moreover, the angiosperms (both EB and DB) showed a similar balanced point near N:Pgr = 15 (14.3 *vs* 15.4 exactly for EB and DB, respectively). Because the mean N:Pgr (18) of EB is greater than 14.3 ($p < 0.05$), the average NRE must be less than PRE (NRE-PRE < 0; Table 1), according to the relative resorption hypothesis (Figure 1). For DB, since its mean N:Pgr (13.5) <15.4 ($p < 0.05$), the average NRE must be larger than PRE (NRE-PRE > 0; Table 1), to satisfy the model we proposed (Figure 1). Our hypothesis can therefore explain the previous inconsistent observations (e.g., [9] *vs* [14] and this study) on patterns of NRE vs. PRE for different plant groups (Table 1).

Conifers had a green-leaf N:P ratio of 9 at NRE = PRE, quite different from that of angiosperms (15). This critical N:Pgr (9) of conifers is consistent with that (9–11) observed in conifer fertilization experiments reported in literature (see Table S2). Similarly, there is evidence for a critical N:Pgr ratio (8) in N-fixing plants. Based on over 400 fertilization experiments worldwide, Sadras (2006) [32] proposed that the optimal N:Pgr ratio of grain legumes achieving maximum yield was 8.7, although for lack of data on fertilization experiments the critical N:P of woody legumes is unclear. Conifers and N-fixers should have a larger average PRE than NRE because of their greater mean foliar N:P (12/23) than the respective assumed critical N:Pgr (9/8) (both $p < 0.05$), according to our model (Figure 1d).

However, as previous studies [8,10,11] suggested, NuRE may be influenced by many factors; these factors include species affiliation (e.g., functional groups, as demonstrated in Table 1 and Figure 1), and site-related variables (e.g., climate, soil nutrient availability). NuRE was found to be significantly correlated with latitude, mean annual temperature, and mean annual precipitation at regional [19] or global scale [9-11]. Soil nutrient availability [5,11,18] and nutrient status of the green-leaves [8,10] were also believed to be important controls on nutrient resorption process. The sampling time may influence the calculation of NuRE, given that the large variations in green-leaf nutrient level within the growing season. The inter-annual variability in NuRE can also be large because of the stresses on plant growth induced by the potential extreme climates. In addition, both ages of the sampled leaves of evergreen plants and plant individuals may affect the green-leaf nutrient concentration and nutrient resorption. All these factors can contribute to the relatively low $r^2$ (0.14-0.30) observed in the inverse relationships between NRE-PRE and green-leaf N:P.

An alternative way to provide support for the hypothesis (represented as the negative relationships in Figure 1) is to calculate the percentage of points in each quadrat of the graph. The major proportion of points in the N:Pgr ~ (NRE-PRE) plane seems to be in their "correct" categories (Figure 1) according to the relative resorption hypothesis. For example, 73% of the points with N:Pgr > 15 are located in the quadrat with NRE-

PRE < 0, and 62% with N:Pgr < 15 are in the quadrat with NRE-PRE > 0, for all species pooled together (Figure 1a). Similarly, about 64%, 50%, and 91% of the points with N:Pgr greater than the assumed corresponding critical ratio (15, 14, and 9, respectively) are located in the quadrat with NRE-PRE < 0, and 74% , 74%, and 40% (2 out of 5 cases) with N:Pgr less than the assumed critical ratios in the quadrat with NRE-PRE > 0, for DB, EB, and conifer, respectively (Figure 1b,c,d). As for the nitrogen fixers, about 80% of the points with N:Pgr greater than the assumed critical ratio (8) are located in the quadrat with NRE-PRE < 0, but there are no points exist with N:Pgr < 8 (i.e., no N-limited cases) (Figure 1d).

As observed by previous studies [5,14], evergreen broadleaves show lower response to nutrient status compared to DB and conifers. The SMA regression slope of (NRE-PRE) is shallower in EB than in the other two plant types (Figure 1), which may be related to the relatively long growing periods and the associated stable rate of nutrient re-translocation in EB.

Indeed, PRE generally showed high sensitivity to nutrient availability (indicated by foliar N:P) across all plant types: the PRE slopes were steeper than NRE slopes. This stronger resorption response to foliar N:P for P *vs* N may also be related to its biochemistry and biogeochemistry (as discussed above on the larger variation in PRE *vs* NRE), and consistent with the hypothesis of stability of limiting elements at the evolutionary level, proposed by Han et al. (2011) [29]. In addition, the sharper response of PRE may suggest that resorption of P seems more important for plant nutrient conservation and N:P stoichiometry in most cases, compared to N, given that the extensive P-limitation in terrestrial plants and large variability in global P availability due to its biogeochemistry [7,28,31,33,34].

Moreover, this inverse relationship of (NRE-PRE) against N:Pgr held true when data were pooled at the vegetation-type level globally, with a critical N:P ratio at about 16 (Figure 5). Both temperate forests in our dataset displayed foliar N:P near the critical value (N/P co-limited), and showed a roughly balanced NRE *vs* PRE. The boreal forests and grasslands had foliar N:P far less than 14 (N-limited), and all showed greater NRE than PRE; while the tropical forests, wetland and desert located in the right lower part of the N:Pgr ~ (NRE-PRE) space, had foliar N:P greater than 16 (P-limited) and PRE > NRE (Figure 5).

Data of boreal and temperate forests in China come from the authors' unpublished data.

Finally, the distinct inverse relationship between foliar N:P and (NRE-PRE) might help deduce the critical (optimal) N:P ratio as illustrated in Figure 1, and to assess the nutritional status (N *vs* P limitation) for a specific community/vegetation or certain plant groups.

### Implication for nutrient cycling across communities with relative limitation

According to our relative resorption hypothesis, when plants are N-limited (e.g., N:P < 10), they are inclined to resorb proportionately more N from senescing leaves, compared to P (NRE-PRE > 0), and correspondingly there will be proportionately less N than P left in senesced leaves. On the contrary, plants tend to resorb proportionally more leaf P, compared to N (NRE < PRE) when they are P-limited (e.g., N:Pgr > 20), and thus leaving proportionately less residual P than N in senesced leaves. The scaling slopes (1.5~1.7 >1) of senesced-leaf N:P against green-leaf N:P are consistent with these predictions across different growth forms (Figure 3).

Since senesced leaves fall and gradually get broken down and decomposed into inorganic nutrients through physical processes and microbial activities, the re-mineralized nutrients from fallen leaves will eventually be taken up by plants (biogeochemical cycling). According to the relative resorption hypothesis, communities with higher average foliar N:P (relative to its corresponding critical N:P) tend to produce litter with disproportionately higher N:P (e.g., EB, conifers, and N-fixing plants; Figure 3), which is a positive feedback for nutrient cycling. For example, if a plant is P-limited (with higher foliar N:P), then year by year the soil will gradually have lower P *vs* N supply (because of the accumulation of litter with much more higher N:P). This situation will exacerbate the P limitation of the plants in the community, which could contribute to the higher PRE than NRE in evergreen broadleaves, conifers, and N-fixing plants. However, this positive feedback of P-limitation can be counteracted and level off through negative feedbacks including the possible disproportionate leaching of "excess" nutrient out of the community (N in such cases), given that litter decomposers may change their carbon-use efficiency to exploit residues with low nutrients [35]. This positive feedback can also be counteracted via shifts in species composition (or, community succession) [33,36,37], and/or the increasing PRE caused by the increasing cost of P-uptake from soil [5]. For communities with lower foliar N:P (e.g., those dominated by deciduous plants; Figure 3b), the converse will be true. Anyway, the difference in decomposability-related traits of the litter (e.g., C:N, lignin:N, and N:P) and the habitat climate (temperature/precipitation) [17], may together contribute to variance in soil nutrient availability, and thus the magnitude of nutrient resorption efficiency (NRE/PRE) in DB *vs* EB *vs* conifers, or in N-fixing *vs* non-N-fixing plants.

These global patterns of decreasing plant foliar and litter N:P with increasing latitude suggest a general tendency of nutrient limitation for plant growth worldwide: N-limitation at middle and high latitudes with a warm to cold and relatively dry climate, and P-limitation at low latitudes with high temperature and precipitation [17,38]. The inverse relation between foliar N:P and (NRE - PRE) demonstrated in our study (Figure 1,5, and Figure S1), can thus better explain the patterns and mechanism of NRE/PRE along the geographical and climatic gradients, which have been observed recently for global plants [9,10]. In fact, the difference between NRE and PRE (i.e., NRE - PRE) did show such latitudinal and climatic patterns (Figure S2).

## Conclusions

Using a global dataset for N and P in both green and senesced leaves, we explored patterns and mechanisms in the responses of NRE *vs* PRE across woody plants with contrasting leaf habits. To our knowledge, this is the first investigation that shows a clear and global-scale negative

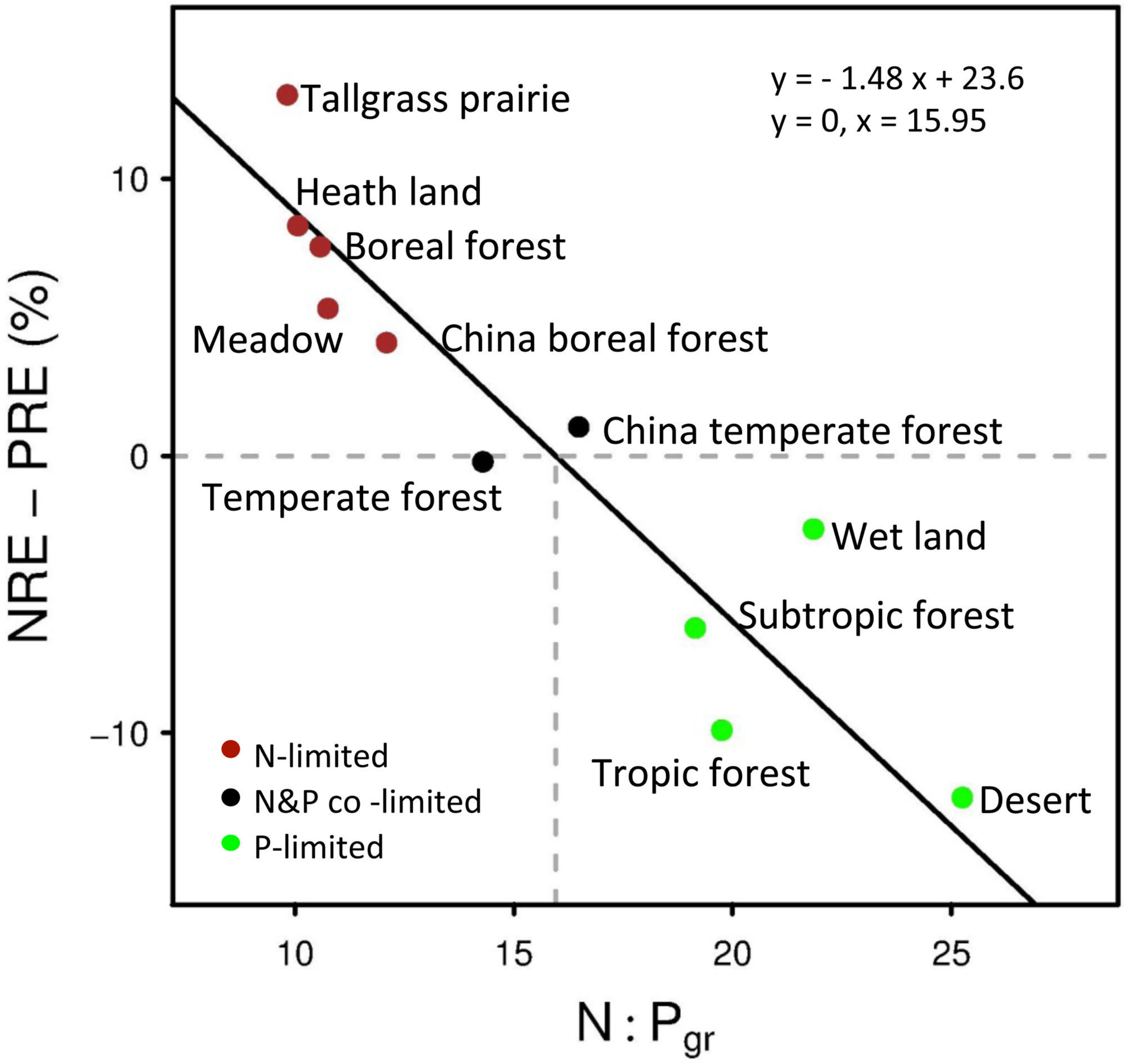


**Figure 5. Figure 5. Standardized major axis regression (solid lines) for the relative resorption efficiency (NRE - PRE) *vs.* green-leaf N:P ratio across different vegetation-types globally.** Data of boreal and temperate forests in China come from the authors' unpublished data.

doi: 10.1371/journal.pone.0083366.g005

relationships between the relative nutrient limitation (based on foliar N:P ratio) and the difference in nutrient resorption efficiency of N *vs* P (indicated by NRE minus PRE) across woody species, growth-forms, and vegetation-types. This relationship supports a relative resorption hypothesis that plants resorb proportionately more of the relatively more limiting nutrient. The relative resorption hypothesis suggests another potential way to determine the critical (optimal) N:P ratio (when NRE ≈ PRE) in a given plant community/ vegetation. Moreover, according to the hypothesis, communities with higher/lower foliar N:P (more likely P/N limited) tend to produce litter with disproportionately higher/ lower N:P, causing a worsening status of P/N availability; this positive feedback may however be counteracted by several negative-feedback mechanisms.

P generally shows higher variability in resorption efficiency, and higher resorption sensitivity to nutrient availability, compared to N, suggesting that the resorption of P seems more important for plant nutrient conservation and N:P stoichiometry. Deciduous plants have lower PRE than NRE

while evergreens, conifers, and N-fixers have higher PRE than NRE, consistent with predictions of the relative resorption hypothesis. Evergreens show lower response of (NRE-PRE) to nutrient status, compared to deciduous plants and conifers, which is possibly related to their relatively long growing seasons and thus the relatively stable rate of nutrient re-translocation in EB.

This study indicates that foliar N:P ratio, together with (NRE-PRE), can provide a useful tool for the assessment of plant N *vs* P nutritional status and improve our understandings of the underlying mechanism of nutrient resorption. This should be helpful in predicting how variations in plant nutrient availability induced by global change will influence the nutrient resorption process in different plant groups and alter global nutrient cycling.

## Supporting Information

**Appendix S1. Literature based on which the dataset were compiled for leaf nitrogen and phosphorus resorption efficiency in global woody plants.**
(PDF)

**Figure S1. Global distribution of green-leaf N:P ratio and the relative resorption efficiency.**
(PDF)

**Figure S2. Relationships between relative resorption efficiency and the latitude/climates.**
(PDF)

**Table S1. Uncorrected nutrient resorption efficiency and senesced-leaf N and P concentration for different plant types.**
(PDF)

**Table S2. Results of fertilization experiments with conifer vegetation, showing green-leaf N:P ratios and nutrient status of the vegetation.**
(PDF)

## Acknowledgements

We thank S. P. Wang, X. Zhao, and L. Jiang for assistance in data collection, X. D. Xie for helpful discussion, and M. J. Lechowicz for constructive comments and language editing. We thank R. P. Niedz and the anonymous reviewers for their valuable comments. The authors also acknowledge all the researchers whose works have contributed to the dataset complied and analyzed in this study.

## Author Contributions

Conceived and designed the experiments: WH JF. Performed the experiments: LT WH. Analyzed the data: WH LT. Contributed reagents/materials/analysis tools: LT YC. Wrote the manuscript: WH LT YC JF.

## References

1. Marschner P (2012) Marschner's mineral nutrition of higher plants. Amsterdam, The Netherland. Academic Press. 651 pp.
2. Chapin FS (1980) The mineral nutrition of wild plants. Annu Rev Ecol Evol Syst 11: 233-260. doi:10.1146/annurev.es.11.110180.001313.
3. Vitousek P (1982) Nutrient cycling and nutrient use efficiency. Am Nat 119: 553-572. doi:10.1086/283931.
4. Killingbeck KT (1996) Nutrients in senesced leaves: Keys to the search for potential resorption and resorption proficiency. Ecology 77: 1716-1727. doi:10.2307/2265777.
5. Wright IJ, Westoby M (2003) Nutrient concentration, resorption and lifespan: Leaf traits of Australian sclerophyll species. Funct Ecol 17: 10-19. doi:10.1046/j.1365-2435.2003.00694.x.
6. Aerts R, Chapin FS (2000) The mineral nutrition of wild plants revisited: A re-evaluation of processes and patterns. Adv Ecol Res 30: 1-67.
7. Güsewell S (2005) Nutrient resorption of wetland graminoids is related to the type of nutrient limitation. Funct Ecol 19: 344–354. doi:10.1111/j.0269-8463.2005.00967.x.
8. Kobe RK, Lepczyk CA, Iyer M (2005) Resorption efficiency decreases with increasing green leaf nutrients in a global data set. Ecology 86: 2780-2792. doi:10.1890/04-1830.
9. Yuan ZY, Chen HYH (2009) Global-scale patterns of nutrient resorption associated with latitude, temperature and precipitation. Glob Ecol Biogeogr 18: 11-18. doi:10.1111/j.1466-8238.2008.00425.x.
10. Vergutz L, Manzoni S, Porporato A, Novais RF, Jackson RB (2012) Global resorption efficiencies and concentrations of carbon and nutrients in leaves of terrestrial plants. Ecol Monogr 82: 205-220. doi:10.1890/11-0416.1.
11. Reed SC, Townsend AR, Davidson EA, Cleveland CC (2012) Stoichiometric patterns in foliar nutrient resorption across multiple scales. New Phytol 196: 173–180. doi:10.1111/j.1469-8137.2012.04249.x. PubMed: 22882279.
12. Agren GI (2008) Stoichiometry and nutrition of plant growth in natural communities. Annu Rev Ecol Evol Syst 39: 153-170. doi:10.1146/annurev.ecolsys.39.110707.173515.
13. Craine JM (2011) Resource strategies of wild plants. Princeton: Princeton University Press.
14. Aerts R (1996) Nutrient resorption from senescing leaves of perennials: Are there general patterns? J Ecol 84: 597-608. doi:10.2307/2261481.
15. Reich PB (1998) Variation among plant species in leaf turnover rates and associated traits: Implications for growth at all life stages. In: H LambersH PorterMMI Van Vuuren. Inherent variation in plant growth. Leiden, The Netherland. Backhuys Publishers. pp. 467-487.
16. Van Heerwaarden LM, Toet S, Aerts R (2003) Nitrogen and phosphorus resorption efficiency and proficiency in six sub-arctic bog species after 4 years of nitrogen fertilization. J Ecol 91: 1060-1070. doi:10.1046/j.1365-2745.2003.00828.x.
17. McGroddy ME, Daufresne T, Hedin LO (2004) Scaling of C : N : P stoichiometry in forests worldwide: Implications of terrestrial redfield-type ratios. Ecology 85: 2390-2401. doi:10.1890/03-0351.
18. Richardson SJ, Peltzer DA, Allen RB, McGlone MS (2005) Resorption proficiency along a chronosequence: Responses among communities and within species. Ecology 86: 20-25. doi:10.1890/04-0524.
19. Lovelock CE, Feller IC, Ball MC, Ellis J, Sorrell B (2007) Testing the growth rate vs. geochemical hypothesis for latitudinal variation in plant nutrients. Ecol Lett 10: 1154-1163. doi:10.1111/j.1461-0248.2007.01112.x. PubMed: 17927772.
20. Sterner RW, Elser JJ (2002) Ecological Stoichiometry: The Biology of Elements from Molecules to the Biosphere. Princeton: Princeton University Press.
21. Koerselman W, Meuleman AFM (1996) The vegetation N:P ratio: A new tool to detect the nature of nutrient limitation. J Appl Ecol 33: 1441-1450. doi:10.2307/2404783.
22. Tessier JT, Raynal DJ (2003) Use of nitrogen to phosphorus ratios in plant tissue as an indicator of nutrient limitation and nitrogen saturation. J Appl Ecol 40: 523-534. doi:10.1046/j.1365-2664.2003.00820.x.
23. Güsewell S (2004) N: P ratios in terrestrial plants: Variation and functional significance. New Phytol 164: 243-266. doi:10.1111/j.1469-8137.2004.01192.x.

24. Van Heerwaarden LM, Toet S, Aerts R (2003) Current measures of nutrient resorption efficiency lead to a substantial underestimation of real resorption efficiency: Facts and solutions. Oikos 101: 664-669. doi: 10.1034/j.1600-0706.2003.12351.x.
25. Sokal RR, Rohlf FJ (1995) Biometry: The principles and practice of statistics in biological research. New York: W.H. Freeman.
26. R Development Core Team ( 2012) R: A language and environment for statistical computing. Vienana, Austria. Available: http://www.r-project.org. Accessed 20 March 2012
27. Oleksyn J, Reich PB, Zytkowiak R, Karolewski P, Tjoelker MG (2003) Nutrient conservation increases with latitude of origin in European Pinus sylvestris populations. Oecologia 136: 220-235. doi:10.1007/s00442-003-1265-9. PubMed: 12756524.
28. Han WX, Fang JY, Guo DL, Zhang Y (2005) Leaf nitrogen and phosphorus stoichiometry across 753 terrestrial plant species in China. New Phytol 168: 377–385. doi:10.1111/j.1469-8137.2005.01530.x. PubMed: 16219077.
29. Han WX, Fang JY, Reich PB, Ian Woodward F, Wang ZH (2011) Biogeography and variability of eleven mineral elements in plant leaves across gradients of climate, soil and plant functional type in China. Ecol Lett 14: 788-796. doi:10.1111/j.1461-0248.2011.01641.x. PubMed: 21692962.
30. Ostertag R (2010) Foliar nitrogen and phosphorus accumulation responses after fertilization: An example from nutrient-limited Hawaiian forests. Plant Soil 334: 85-98. doi:10.1007/s11104-010-0281-x.
31. Townsend AR, Cleveland CC, Asner GP, Bustamante MMC (2007) Controls over foliar N : P ratios in tropical rain forests. Ecology 88: 107-118. Available online at: doi: 10.1890/0012-9658(2007)88[107:COFNRI]2.0.CO;2. PubMed: 17489459
32. Sadras VO (2006) The N:P stoichiometry of cereal, grain legume and oilseed crops. Field Crops Res 95: 13–29. doi:10.1016/j.fcr.2005.01.020.
33. Vitousek PM, Porder S, Houlton BZ, Chadwick OA (2010) Terrestrial phosphorus limitation: mechanisms, implications, and nitrogen–phosphorus interactions. Ecol Appl 20: 5-15. doi:10.1890/08-0127.1. PubMed: 20349827.
34. Menge DNL, Hedin LO, Pacala SW (2012) Nitrogen and phosphorus limitation over long-term ecosystem development in terrestrial ecosystems. PLOS ONE 7: e42045. doi:10.1371/journal.pone.0042045. PubMed: 22870281.
35. Manzoni S, Jackson RB, Trofymow JA, Porporato A (2008) The global stoichiometry of litter nitrogen mineralization. Science 321: 684-686. doi:10.1126/science.1159792. PubMed: 18669860.
36. Tilman D (1982) Resource competition and community structure. Monogr Popul Biol 17: 1-296.
37. Tilman D (1987) Secondary succession and the pattern of plant dominance along experimental nitrogen gradients. Ecol Monogr 57: 189-214. doi:10.2307/2937080.
38. Reich PB, Oleksyn J (2004) Global patterns of plant leaf N and P in relation to temperature and latitude. Proc Natl Acad Sci U S A 101: : 11001-11006. doi:10.1073/pnas.0403588101. PubMed: 15213326.
